# Management of Intracranial Metastases in EGFR-Mutated NSCLC: A Review of Literature following an Unusual Case Report

**DOI:** 10.1155/2021/5526809

**Published:** 2021-07-02

**Authors:** Víctor Albarrán, Javier Pozas, Juan José Soto, Jorge Esteban, Elena Corral, Yolanda Lage, Pablo Gajate, Pilar Garrido

**Affiliations:** Thoracic Tumors Unit from Medical Oncology Department, Ramón Y Cajal University Hospital, Madrid, Spain

## Abstract

The arrival of subsequent generations of tyrosine-kinase inhibitors (TKIs) has significantly broaden the EGFR-mutated lung cancer therapeutic landscape. Results from the FLAURA clinical trial have pushed osimertinib to the first-line treatment for patients with advanced-stage disease, showing outstanding control rates of intracranial metastases, considerably higher than those of the first and second-generation EGFR TKIs. A progressively better knowledge of short and long-term neurocognitive side effects of radiotherapy, as well as the lack of evidence about the benefit of its combination with TKIs, has opened a debate about its indication at diagnosis of intracranial disease, at least before the response to targeted therapy has been evaluated. However, there is a small percentage of primarily resistant cases to osimertinib, mainly due to histologic transformation, acquired EGFR mutations and off-target genetic resistances that lead to a scenery of poor clinical prognosis in which radiotherapy may have a higher relevance for the management of brain metastases. We offer a review of the current recommendations for the management of intracranial metastases in EGFR-mutated NSCLC and the resistance mechanisms to third-generation TKIs, following the report of an unusual clinical case with a rapid progression to osimertinib.

## 1. Introduction

Lung cancer continues to be one of the first causes of cancer-related deaths around the world, being non-small-cell lung cancer (NSCLC) the most frequent histological subtype (85% of the cases) and presenting in over 80% of the patients at an advanced stage of disease (IIIB-IV).

Brain metastases are associated with a poor prognosis and significantly reduce overall survival (OS), being more prevalent in tumors that express somatic mutations of epidermal growth factor receptors (EGFR).

Over the last few years, the development of successful therapies against a wide spectrum of molecular targets, including the approval of multiple generations of EGFR tyrosine-kinase inhibitors (TKIs), has dramatically changed the therapeutic approach to NSCLC.

Due to the difficulty for conventional drugs to cross the blood-brain barrier, radiotherapy has been for years the preferred treatment strategy for NSCLC with brain metastatic disease, despite a significant rate of neurological toxicity resulting in a nonnegligible morbidity.

Promising data regarding TKI effectiveness, including their intracranial disease control rate, have led the international scientific community to discuss the current role of radiotherapy in this subpopulation of patients.

## 2. Case Presentation

Our patient is a 58-year-old nonsmoker woman with no relevant previous medical history, who was admitted to the hospital emergency department after an episode of disorientation following a complex partial seizure. She had been previously asymptomatic except for a several-weeks long minor headache, and physical examination only revealed mild bradypsychia and tangential speech, without any further focal neurological symptom.

**Figure 1:**
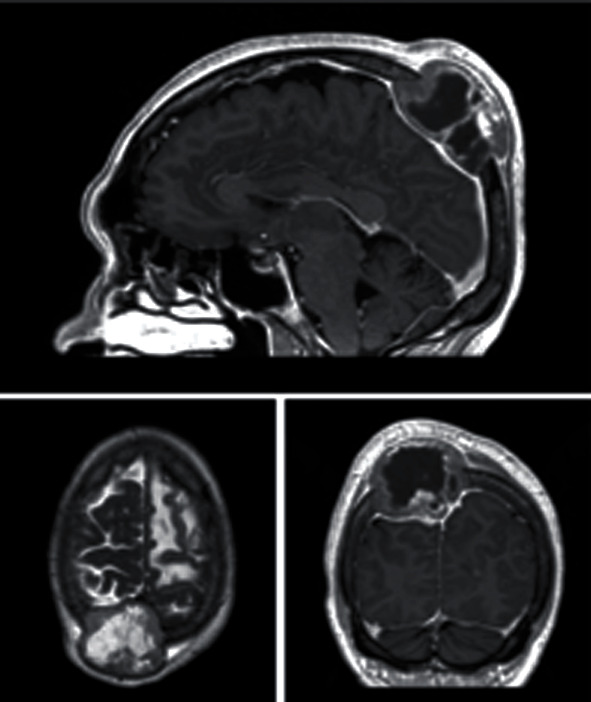
Images from brain MRI study at diagnosis.

Brain CT scan was performed, displaying a bulky lesion emerging from the parietal bone. This finding was later confirmed by brain MRI, showing a direct contact between the lesion and dura mater, as well as an intravascular tumor thrombus in the superior sagittal sinus (Figure 1).

An additional 18 mm intracranial lesion was identified in the left frontal lobe, associated with an incipient brain midline shift due to significant perilesional edema, together with at least four smaller brain metastases (<10 mm).

**Figure 2:**
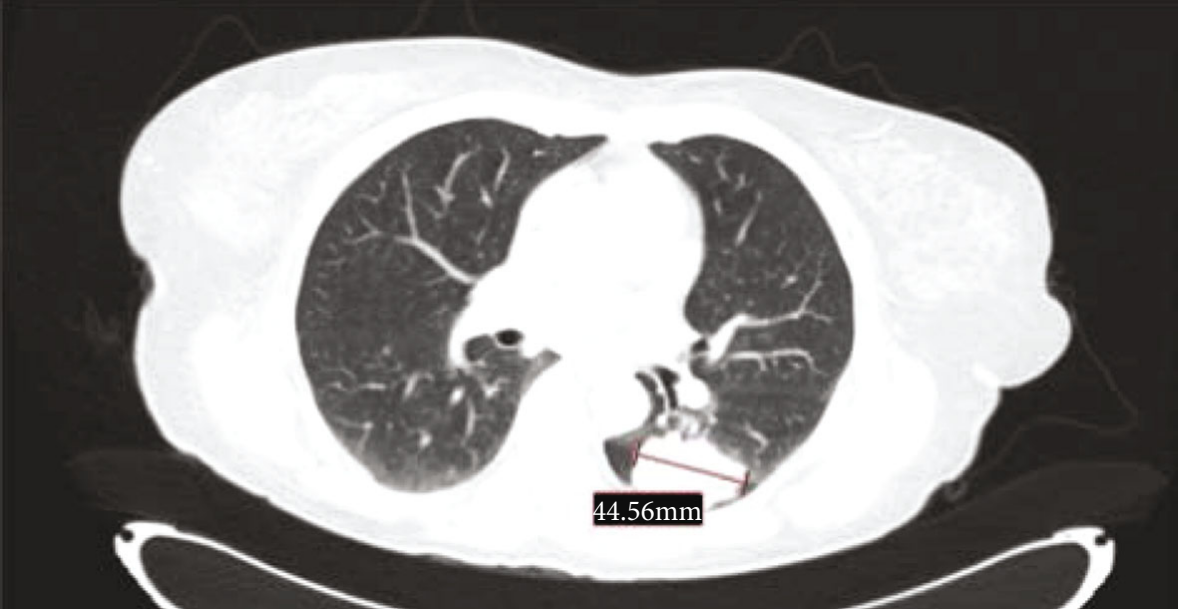
Lung mass on thoracic CT scan at diagnosis.

An extensive body workup was performed, revealing a 44 mm lung mass on the left lower lobe, as well as mediastinal, supraclavicular and axillary lymph nodes, metastatic lesions in the left kidney, left adrenal gland, and left iliac bone (stage cT2bN3M1c) (Figure 2).

A large-core needle biopsy of a supraclavicular lymph node was compatible with an EGFR-mutated lung adenocarcinoma (exon 19 deletion). Following current treatment guidelines, first-line therapy with third-generation EGFR inhibitor osimertinib was initiated, with an adequate tolerance and no immediate toxicity. Brain radiotherapy was deferred after the case was discussed in the multidisciplinary tumor board, given the molecular profile of the tumor and the possibility of using targeted therapy.

**Figure 3:**
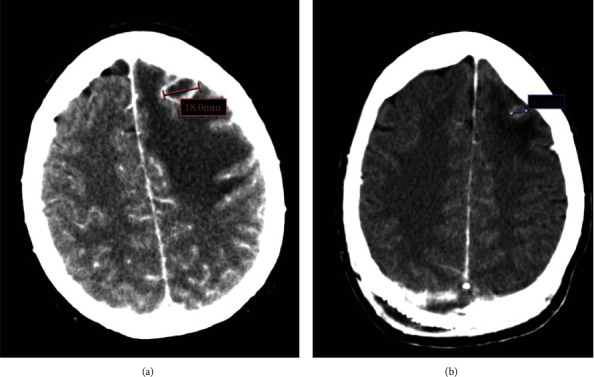
Image of left frontal lobe lesion from CT scan at diagnosis (a) and reevaluation CT scan performed six weeks after the initiation of osimertinib (b), showing a partial response (from 18 mm to 8 mm).

High-dose steroids (dexamethasone 4 mg/8 h) and anticonvulsant treatment with levetiracetam were initiated. Given the risk of dura mater external exposure, palliative excisional surgery was performed. The patient presented a favorable clinical outcome and was later discharged from the hospital. Partial response of the other intracranial lesions was confirmed by early brain CT scan reevaluation (Figure 3).

Nevertheless, three months after hospital discharge, the patient was admitted at the emergency department after a new episode of disorientation and decreased conscious level. A CT scan was performed, showing transtentorial and subfalcine herniation due to progressive intracranial disease with intense perilesional edema. After moderate clinical improvement with high-dose steroids, the patient received stereotactic body radiation therapy (SBRT) and initiated and a second-line treatment with platinum-based chemotherapy.

Progression to chemotherapy was confirmed after three months, and since further active treatment was contraindicated due to significant clinical deterioration, the patient settled in a palliative care center to receive the best supportive care, passing away soon afterwards.

## 3. Discussion

Nearly 25% of patients with EGFR-mutated NSCLC present intracranial metastasis at diagnosis, increasing to 45% after 3 years from debut [1]. Brain disease is a potential major complication that decreases median overall survival to 7-12 months and significantly impacts on global quality of life.

Over the past few years, therapeutic management of EGFR-mutated NSCLC has been profoundly transformed by successive generations of tyrosine-kinase inhibitors (TKIs). First-generation TKIs (erlotinib and gefitinib) entailed a significant survival improvement when compared to platinum-based chemotherapy, reaching response rates of 56-74% and a median progression-free survival (PFS) of 10-14 months [2].

However, most patients relapsed within the first two years of treatment due to acquired resistance mechanisms, being the most frequent one (50-60%) an additional mutation on EGFR (T790) that increases ATP affinity and triggers molecular pathways that decrease TKI effectiveness [3].

A new generation of irreversible TKIs (afatinib and dacomitinib), despite their promising results in preclinical models, attained response rates below 10% and PFS below 4 months in patients pretreated with first-generation TKIs, probably due to the difficulty of inhibiting T790M at a clinically tolerable dosage [4].

Third-generation TKIs began to arise in this context, with a powerful inhibitory activity over T790M and EGFR sensitivity mutations. In November 2015, osimertinib received FDA approval for the treatment of EGFR-T790M mutated NSCLC, according to data from phase II clinical trial AURA, which showed its efficacy after disease progression to first- and second-generation TKIs [5]. According to final phase III AURA3 overall OS analysis, no statistically significant benefit in OS was observed for osimertinib versus platinum-pemetrexed as a second-line treatment, although this possibly reflects a high crossover rate from chemotherapy to the TKI group [6].

Direct comparison of osimertinib vs. erlotinib and gefitinib in TKI-naïve patients (FLAURA trial) showed a significant clinical benefit with a PFS increase of 8.2 months (18.9 vs. 10.2) and an OS increase of 6.8 months (38.6 vs. 31.8, HR 0.799) [7]. These results allowed osimertinib to become a new *standard of care* in first-line treatment for patients with advanced EGFR-mutated NSCLC.

Erlotinib and gefitinib effectiveness on brain metastases reaches response rates around 36.5% and a median PFS of 11.2 months, with no reported case of complete radiological response in patients that received TKIs alone (without concomitant radiotherapy) [8].

Surprisingly, osimertinib has an outstanding efficacy on nervous central system, reaching intracranial response rates between 54% [9] and 70% [10], with several reported cases of complete remission in patients that never received radiotherapy and irrespective of the number of lesions.

Data from FLAURA trial, collected from 128 patients with brain metastases, showed a clear advantage for osimertinib, with a not reached median intracranial PFS at time of analysis (16.5 months) vs. 13.9 months for the control group with a first-generation TKI (HR 0.48).

Osimertinib had a global response rate of 91% in patients with brain metastases (versus 68% for erlotinib), and this benefit was observed irrespective of previous radiotherapy treatment. Furthermore, patients that received osimertinib as first-line treatment had a better intracranial response than those who received it after disease progression to first or second-generation TKIs.

Although radiotherapy has been the most effective approach to unresectable intracranial metastases for decades, its wide spectrum of toxicity is well known, entailing both acute neurological symptoms (somnolence, headache, or worsening of previous focal deficits), subacute encephalopathy secondary to diffuse demyelination, and late effects that generally appear after 6 months from treatment initiation, such as intracranial hypertension and severe neurocognitive decline.

Twenty-nine studies including 748 adults that received therapeutic cranial irradiation (TCI) and eighteen studies of prophylactic cranial irradiation (PCI) with 368 patients were reviewed in a meta-analysis, showing significant adverse outcomes in terms of cognitive and emotional functioning in 213 TCI patients (28.5%) and 100 PCI patients (27.2%) [11].

A better knowledge of brain radiation side effects and the rapid improvement of targeted therapies—reaching intracranial response rates that match or even surpass those of radiotherapy—have unavoidably led scientific community to reconsider its benefit for NSCLC with *driver* mutations.

Up to date, radiotherapy treatment prior to osimertinib initiation has not shown to increase response rates, PFS, or OS in patients with EGFR-mutated NSCLC [12].

Some authors have hypothesized that TKIs might lead to tumor radiosensitization and also that radiotherapy may facilitate TKIs arrival to the central nervous system through increasing the blood-brain barrier permeability. Meta-analysis and retrospective studies performed so far have shown ambivalent results, being some of them favorable to the use of radiotherapy alone or combined with TKIs [13], whereas others support the use of TKIs in monotherapy [14, 15], perpetuating the discussion about the most appropriate strategy for this subgroup of patients. Nevertheless, it is worth commenting that the above-mentioned studies used only first- and second-generation TKIs as active comparators; hence, new studies with osimertinib are awaited in order to further address this topic.

To our knowledge, there is just one phase III trial (BRAIN [16]) that compares the efficacy and safety of EGFR-TKIs (icotinib) vs. radiotherapy (in combination with platinum-based chemotherapy), with a scarce benefit for the second group. Although direct comparison between radiotherapy and osimertinib alone has not been done yet, given its clear superiority to previous TKIs in terms of intracranial activity, a negligible or even insignificant clinical benefit is expected from combining radiotherapy to new-generation TKIs.

Taking into account the low incidence of intracranial disease progression in patients that receive osimertinib as first-line treatment, the rapid lesion shrinkage (median of 6 months in FLAURA experimental group), and the relevant late toxicity that radiotherapy may cause in patients with increasing survival rates, it seems reasonable to delay brain irradiation at least until initial response to osimertinib has been evaluated.

Every EGFR-mutated NSCLC eventually develops resistance to TKIs that can be essentially grouped into EGFR-dependent and EGFR-independent mechanisms. Data from AURA3 and FLAURA studies indicate different resistance patterns according to treatment line.

However, most of the available data on resistance mechanisms to osimertinib were obtained through plasma genotyping analysis that misses histological transformation rate and has a lower sensitivity for copy number changes and gene fusions [17].

Schoenfeld et al. showed that a small percentage of EGFR-mutated lung tumors are primarily resistant to osimertinib, with very short or even lacking disease response, mainly due to squamous histological transformation, acquired EGFR mutations (such as *G724S*) and off-target genetic resistance (MET amplifications, KRAS mutation, RET, or BRAF fusions) [18]. These emerging primary resistance mechanisms to osimertinib could explain the rapid disease progression in our patient and are associated with poor clinical outcomes, a context in which radiotherapy may have a higher relevance in the management of intracranial metastatic disease.

## 4. Conclusion

Third-generation TKI osimertinib has shown high response rates of intracranial metastases from EGFR-mutated NSCLC, what together with a better recognition of short- and long-term neurocognitive toxicity derived from radiotherapy has raised some doubts about the indication of early brain irradiation for this subgroup of patients. The existence of a significant percentage of tumors that are primarily resistant to EGFR-TKIs, usually entailing poor clinical outcomes, is an emerging matter of concern. Further research is needed for a better characterization of the mechanisms of resistance to osimertinib, the finding of predictive biomarkers of TKI failure, and the development of novel therapeutic strategies.
